# The vesicular trafficking system component MIN7 is required for minimizing *Fusarium graminearum* infection

**DOI:** 10.1093/jxb/erab170

**Published:** 2021-04-20

**Authors:** Ana K Machado Wood, Vinay Panwar, Mike Grimwade-Mann, Tom Ashfield, Kim E Hammond-Kosack, Kostya Kanyuka

**Affiliations:** 1 Biointeractions and Crop Protection, Rothamsted Research, Harpenden AL5 2JQ, UK; 2 Crop Health and Protection (CHAP), Rothamsted Research, Harpenden AL5 2JQ, UK; 3 Queen’s university, Canada

**Keywords:** Arabidopsis, *AtMin7*, disease resistance, fungal pathogenicity, *Fusarium graminearum*, vesicular trafficking, VIGS, wheat

## Abstract

Plants have developed intricate defense mechanisms, referred to as innate immunity, to defend themselves against a wide range of pathogens. Plants often respond rapidly to pathogen attack by the synthesis and delivery to the primary infection sites of various antimicrobial compounds, proteins, and small RNA in membrane vesicles. Much of the evidence regarding the importance of vesicular trafficking in plant–pathogen interactions comes from studies involving model plants whereas this process is relatively understudied in crop plants. Here we assessed whether the vesicular trafficking system components previously implicated in immunity in Arabidopsis play a role in the interaction with *Fusarium graminearum*, a fungal pathogen well-known for its ability to cause *Fusarium* head blight disease in wheat. Among the analysed vesicular trafficking mutants, two independent T-DNA insertion mutants in the *AtMin7* gene displayed a markedly enhanced susceptibility to *F. graminearum*. Earlier studies identified this gene, encoding an ARF-GEF protein, as a target for the HopM1 effector of the bacterial pathogen *Pseudomonas syringae* pv. *tomato*, which destabilizes MIN7 leading to its degradation and weakening host defenses. To test whether this key vesicular trafficking component may also contribute to defense in crop plants, we identified the candidate *TaMin7* genes in wheat and knocked-down their expression through virus-induced gene silencing. Wheat plants in which *TaMin7* genes were silenced displayed significantly more Fusarium head blight disease. This suggests that disruption of MIN7 function in both model and crop plants compromises the trafficking of innate immunity signals or products resulting in hypersusceptibility to various pathogens.

## Teaser

Disruption of the ARF-GEF protein encoding gene *AtMin7* in Arabidopsis and silencing of the orthologous gene in wheat result in hypersusceptibility to the fungal pathogen *Fusarium graminearum*.

## Introduction

In nature, plants are frequently exposed to various environmental stresses including pathogens, and yet more often than not plants appear healthy or show only weak or mild disease symptoms. To maintain this healthy status, plants have evolved an elaborate and tightly regulated innate immune system that allows them to restrict pathogen invasion or slow down/minimize disease progression (Jones and Dangl, 2006). These defensive processes not only include secretion of various peptides and secondary metabolites in response to pathogen attack (Dixon, 2001; van Loon *et al.*, 2006; Yun *et al.*, 2013), but also the tight regulation of this secretion (Yun *et al.*, 2013).

In addition to the cell wall, each plant cell is enclosed by the plasma membrane, and the cytoplasmic contents include a variety of membrane-enclosed organelles. Transport of various cargo molecules across different membranes and the sorting of these into the correct cellular compartments is a process central to the functioning of multiple plant cell types (Bassham *et al.*, 2008). The transport of components in small, membrane-bound vesicles between the intra- and extracellular space is referred to as vesicular trafficking (Yun and Kwon, 2017). Regulation of multiple cellular responses by the membrane trafficking network during plant–microbe interactions is required to facilitate a coordinated defense response at sites of pathogen attack (Ben Khaled *et al.*, 2015; Gu *et al.*, 2017; Yun and Kwon, 2017; Ekanayake *et al.*, 2019).

**Fig. 1.**
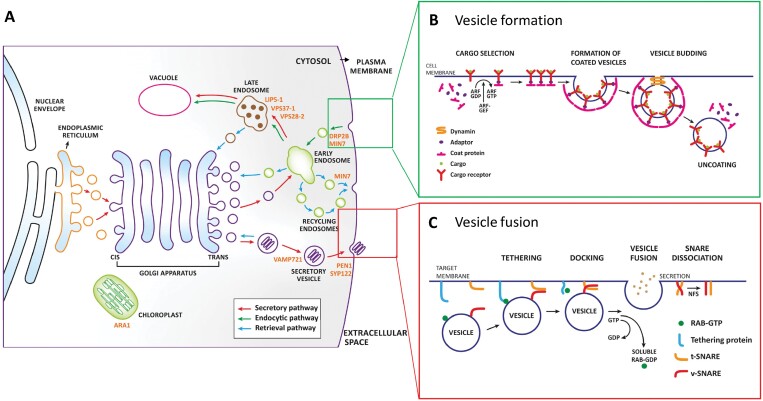
Schematic diagram of vesicular trafficking pathways in Arabidopsis. (A) Major cellular vesicular trafficking pathways: secretory (red arrows), endocytic (green arrows) and retrieval (blue arrows). Labels in orange indicate the location of specific regulators of the vesicular trafficking system. (B) Vesicle formation and budding. Bud formation requires small GTPases of ADP-ribosylation factor (ARF) or secretion-associated Ras-related protein (SAR1) type as well as adaptor proteins that recognize and recruit specific cargo receptors. Phosphorylation and activation of ARF/SAR1 are regulated by guanine nucleotide exchange factor (GEF) proteins that stimulate the release of GDP to allow binding of GTP. The latter triggers a conformational change in ARF/SAR1 that allows their stable association with the membrane surface and recruitment of specific coat proteins (COP), initiating the budding process. Once the rounded vesicle shape is formed, the large dynamin-related multidomain GTPases catalyse membrane scission, generating a transport vesicle. The coat components are rapidly lost shortly after the vesicle buds off. (C) Fusion of vesicles with the correct target membranes. Fusion of vesicles to the correct target membranes is regulated by the small Rab GTPase proteins. Different Rab proteins are associated with one or more membrane-enclosed organelles of the secretory pathway. Once in the GTP bound state, the Rab GTPase proteins bind to specific tethering factors in the target membrane to establish the first connection between the membranes that are going to fuse. Next, the *N*-ethylmaleimide-sensitive factor adaptor protein receptor (SNARE)-domain containing proteins on both the vesicle and the targeting membrane dock to mediate the fusion of the two membranes. SNAREs are transmembrane proteins that exist in complementary sets. Those located in the vesicle are called v-SNAREs or R-SNAREs, and those located in the target membrane are known as t-SNAREs or Q-SNAREs. A trans-SNARE complex formed following binding of v-SNARE/R-SNARE to t-SNARE/Q-SNARE located on separate membranes catalyses the membrane fusion (Collins *et al.*, 2003; Yun and Kwon, 2017). Figure based on Alberts *et al.* (1989).

The vesicular trafficking system comprises two main pathways, secretory and endocytic (Fig. 1A), with both implicated in effective immunity against pathogens. A wide range of defense-related proteins, antimicrobial metabolites, and compounds such as callose that strengthen the plant cell wall can potentially be secreted at the sites of pathogen invasion (Gu *et al.*, 2017). Concomitantly cell-surface immune receptors are subjected to endocytosis, which is necessary for initiation of signal transduction and regulation of receptor activity (e.g. through recycling or degradation in the vacuole) (Ranf, 2017). Secretory pathways transport newly synthesized proteins and other macromolecules (collectively referred to as ‘cargo’) from the endoplasmic reticulum via the Golgi apparatus to the plasma membrane or the extracellular space (Bassham *et al.*, 2008). In the endocytic pathway, membranous vesicles internalized in the plasma membrane undergo homotypic fusion to form early/sorting endosomes. Internalized cargo can then be sorted and recycled back to the plasma membrane through the recycling endosome, sent to the *trans-*Golgi network (TGN) via retrograde trafficking mechanisms, or trafficked through the late endosome/multivesicular body to the vacuole (Fig. 1A) (Kwon *et al.*, 2008*a*, *b*; Dodds and Rathjen, 2010; Bozkurt *et al.*, 2011; Nomura *et al.*, 2011; Ellinger *et al.*, 2013).

The increasing number of mutations in plant vesicular trafficking genes that show altered resistance to microbial pathogens illustrates the importance of vesicular trafficking to plant immunity (Rybak and Silke, 2019). Whereas the general understanding of these trafficking pathways has advanced, knowledge of the molecular mechanisms employed by plant cells to coordinate the transport of specific molecules through different cell compartments is still limited (Ekanayake *et al.*, 2019). The components of vesicular trafficking do not function in isolation but form complexes. Within these complexes, a defined set of proteins are responsible for selecting and transporting specific cargo molecules in a way that is highly coordinated both temporally and spatially (Fig. 1B, C) (Rutter and Innes, 2018).

Plant pathogens are known to secrete small effector proteins some of which could specifically target components of vesicular trafficking important for plant immunity. One of these targets is the regulator protein adenosine diphosphate ribosylation factor–guanine nucleotide exchange factor (ARF-GEF) encoded by *AtMin7/Ben1/Big5* in Arabidopsis. The bacterial effector HopM1, which is required for proliferation of *Pseudomonas syringae* pv. *tomato* in the apoplast of the leaf interior, has been shown to degrade MIN7 in Arabidopsis (Nomura *et al.*, 2006). ARF-GEF proteins are involved in controlling vesicle formation by regulating the ARF-family of small GTPases (Singh and Jürgens, 2018). Another example is the *Phytophthora infestans* effector AvR3a, which perturbs pattern-triggered immunity responses partly by targeting Dynamin-Related Protein 2B (DRP2B) in Arabidopsis (Chaparro-Garcia *et al.*, 2015). Dynamins are important regulators of clathrin-mediated endocytosis and are involved in the scission and release of clathrin-coated vesicles from the plasma membrane (Gu *et al.*, 2017).

Much of the evidence regarding the role of vesicular trafficking in plant immunity comes from studies involving the model plants Arabidopsis and *Nicotiana benthamiana* and to a lesser extent crop species such as barley (*Hordeum vulgare*), and a small number of their respective adapted biotrophic or hemi-biotrophic bacterial (*P. syringae*), oomycete (*P. infestans*) or ascomycete powdery mildew pathogens (*Golovinomyces cichoracearum*, *Blumeria graminis*, and *Erysiphe pisi*) (Nomura *et al.*, 2006; An *et al.*, 2006; Wang *et al.*, 2009; Schmidt *et al.*, 2014; Chaparro-Garcia *et al.*, 2015). However, little is still known about the role of plant vesicular trafficking in the interactions involving other pathogens, other crop species, and those that primarily infect non-leaf tissue.

Fusarium head blight (FHB) disease, caused by the ascomycete fungus *Fusarium graminearum* and related *Fusarium* species, causes substantial yield losses and reduced grain quality and safety in a number of major cereal crops, such as wheat, barley, maize, rye, triticale, and oat, worldwide (McMullen *et al.*, 2012; Dean *et al.*, 2012). Moreover, under laboratory conditions *F. graminearum* is able to infect floral tissue of intact Arabidopsis plants as well as detached leaves (Chen *et al.*, 2006; Cuzick *et al.*, 2008; Brewer and Hammond-Kosack, 2015; Wood *et al.*, 2020). For this model plant species, a large mutant collection is readily available.

The aim of this study was to assess whether knock-out mutations of the individual components of the vesicular trafficking system in Arabidopsis previously implicated in plant immunity had any impact on the interaction with *F. graminearum*. Screening of the assembled collection of mutants using a detached-leaf bioassay identified two independent T-DNA insertion mutants in the *AtMin7* gene, which displayed striking hyper-susceptibility to *F. graminearum* strain PH-1 compared with the parental wild-type Arabidopsis ecotype Col-0. Utilizing a recently released high-quality fully annotated wheat genome reference sequence assembly (International Wheat Genome Sequencing Consortium, 2018) and well-established bioinformatics tools enabling identification of putative gene orthologs from different plant species (Adamski *et al.*, 2020), we identified the three homoeologous *TaMin7* genes in hexaploid wheat (*Triticum aestivum*). Knock-down of these genes using virus-induced gene silencing (VIGS) (Lee *et al.*, 2012) significantly promoted FHB disease formation in this crop species.

## Materials and methods

### Plant material and growth conditions

The Arabidopsis mutants (Table 1) and the corresponding wild-type parental ecotype Col-0 used in the study were obtained from the Nottingham Arabidopsis Stock Centre. The corresponding mutations were verified by PCR using the primers listed in Supplementary Table S1 and confirmed homozygous mutants selected for further study. Arabidopsis seeds were sown in Levington F2+S compost (Everris Ltd) and stratified in the dark for 4 d at 5°C before transferring to a controlled environment growth chamber operating at 20°C/17°C during day/night with a 12-h photoperiod (light intensity of approximately 80–100 µmol m^−2^ s^−1^).

**Table 1.** Arabidopsis mutants used in this study.

Gene name	Gene ID	Gene description	Mutant name	Mutant accession No.	Reference	Evidence in Arabidopsis plants
*Ara1/AtRabA5E*	At1g05810	Arabidopsis gene homologous to the Ras oncogene 1/Rab GTPase-like A5A	*ara1*	SM_3.15870	Karim *et al.* (2014)	Interacts with proteins involved in multiple stress responses
*Drp2b*	At1g59610	Dynamin-related protein 2B	*drp2b-1*	SALK_003049	Smith *et al.* (2014)	Functions as a novel component of flagellin-mediated defense responses against bacterium *P. syringae*
			*drp2b-2*	SALK_134887		
*Pen1/ Syp121*	At3g11820	Penetration resistance 1/Syntaxin of plants 121	*pen1*	SALK_004484C	Collins *et al.* (2003), Johansson *et al.* (2014)	Absence of Pen1 is associated with increased tissue penetration by the non-adapted fungal pathogen *Blumeria graminis* f.sp. *hordei*
*Syp122*	At3g52400	Syntaxin of plants 122	*syp122-1*	SALK_008617	Zhang *et al.* (2007)	Acts together with SYP121 as a negative regulator of programmed cell death, salicylic acid, jasmonic acid, and ethylene pathways
*AtMin7/ Ben1/* *Big5*	At3g43300	HopM1 interactor 7/BFA-visualized endocytic trafficking defective 1/Brefeldin A-inhibited guanine nucleotide-exchange protein 5	*atmin7-1*	SALK_012013	Nomura *et al.* (2006)	Encodes an immunity-associated ARF-GEF family protein targeted by HopM1, conserved bacterial *P. syringae* pv. *tomato* virulence protein
			*atmin7-2*	SALK_013761		
*Vps37*	At3g53120	Vacuolar protein sorting 37	*vps37-1*	SALK_042859C	Spallek *et al.* (2013)	Co-localizes with FLS2 (FLAGELLIN SENSING 2) immune receptor at endosomes and immuno-precipitates with this receptor upon flg22 elicitation
*Vps28*	At4g05000	Vacuolar protein sorting 28	*vps28-2*	SALK_040274	Spallek *et al.* (2013)	Critical for immunity against bacterial infection through a role in stomatal closure; regulates late FLS2 endosomal sorting
*Vamp721*	At1g04750	Vesicle-associated membrane protein 721	*vamp721*	SALK_037273C	Kwon *et al.* (2008*b*), Kim *et al.* (2014)	Forms a complex with PEN1; mediates trafficking of the powdery mildew resistance protein RPW8.2 to the extrahaustorial membrane of haustorial complexes
*Lip5*	At4g26750	LYST-interacting protein 5	*lip5-1*	SAIL_854_F08	Wang *et al.* (2014)	Disruption of Lip5 causes increased susceptibility to the bacterial pathogen P. syringae

The fully susceptible spring type bread wheat (*Triticum aestivum*) cv. Bobwhite was used in this study (Cuzick *et al.*, 2008). The plants were grown in a controlled-environment growth chamber with day/night temperatures of 23°C/20°C at around 65% relative humidity and a 16-h photoperiod with light intensity of approximately 180 µmol m^−2^ s^−1^.

### Fungal growth conditions, plants inoculation, and disease assessment

The reference *F. graminearum* strain PH-1 was used in this study. Routine culturing of the fungus, conidiospore induction, and preparation of conidial suspensions followed essentially the same procedures as described in Brown *et al.* (2011). Conidiospore suspensions harvested in sterile distilled water were adjusted to a concentration of 5×10^5^ or 1×10^5^ conidia ml^−1^ for inoculation of Arabidopsis or wheat, respectively.

Detached Arabidopsis leaves were inoculated as described in Chen *et al.* (2006) with the following modification. Fully expanded rosette leaves were detached from the 6-week-old plants using razor blades and placed adaxial surface facing upwards on the surface of 1% water agar in 10×10 cm square sterile Petri dishes, with eight leaves per dish. Each leaf was then superficially wounded by gently puncturing over the midrib with a glass Pasteur pipette and a 5 µl droplet of *F. graminearum* conidiospore suspension supplemented with 20 µM deoxynivalenol (DON) was deposited onto the fresh wound. Mock inoculation was carried out using a 5 µl droplet of sterile distilled water supplemented with 20 µM DON. After inoculation, the plates were transferred to the controlled-environment growth chamber operating at 20°C/17°C during day/night and 16-h photoperiod but kept in the dark for the first 3 d following which plates were incubated under low light (40 µmol m^−2^ s^−1^) for a further 4 d before the disease assessment took place.

Color (RGB) photographs were taken at 6 d after inoculation using a Nikon (D90) camera and backlighting to ensure consistent illumination. Image analysis to quantify the diseased areas was conducted using the LemnaTec LemnaGrid software module (LemnaTec GmbH, Aachen, Germany). Leaf areas were segmented using a combination of a color-based classification and thresholding after converting the images to grayscale. Filters were applied to remove misclassified pixels and to fill in gaps. Finally, a customized color-based classification was applied to score leaf-area pixels as belonging to diseased or healthy tissue.

Intact spikes of adult wheat cv. Bobwhite plants were point inoculated at the first signs of anther extrusion by depositing 5 μl of conidial suspension in the floral cavity between the palea and lemma of the outer two florets located in the upper one-third of the spike, as previously described (Brown *et al.*, 2011). Control plants were inoculated with sterile water only. Inoculated plants were incubated in a humid chamber for 48 h of which the first 24 h were in darkness. The inoculated plants were then kept in a controlled-environment growth chamber at approximately 65% humidity, the progression of the disease was visually monitored every 3 d, and the number of bleached spikelets below the inoculated spikelet on each spike was recorded along with the total number of spikelets on each spike (Urban *et al.*, 2003).

### PCR-based confirmation of specific mutations in the T-DNA insertion mutant Arabidopsis plants obtained from the seed stock center

One leaf from each Arabidopsis plant was collected in a 2 ml micro-tube, frozen in liquid nitrogen, and then ground with a micro-pestle. DNA extraction was carried as described in Motteram *et al.* (2009). Briefly, ground tissue was added to 350 μl of TEN buffer (500 mM NaCl, 400 mM Tris–HCL, 50 mM EDTA, pH 8.0), 1% β-mercaptoethanol, 5 mM 1,10-phenanthroline, and 2% (w/v) polyvinylpyrrolidone (K30). The resulting suspension was then mixed thoroughly with 350 μl of 2% (w/v) SDS and incubated for 30 min at 65°C. Following the addition of 300 μl of ice-cold ammonium acetate (7.5 M), the sample was kept on ice for 20 min and then centrifuged at 15 000 *g* for 10 min. DNA was precipitated with isopropanol, washed with 70% (v/v) ethanol, and dissolved in sterile distilled water.

For genotyping an insertion line using three primers in two combinations pairs, we carried out two PCR reactions: the ‘wild-type PCR’ and the ‘T-DNA PCR’ (O’Malley *et al.*, 2015). Primer sequences were available at the Salk Institute Genomic Analysis Laboratory (SIGnAL) database and are listed in Supplementary Table S1. The wild-type PCR reaction used gene-specific ‘right primer’ (RP) and ‘left primer’ (LP) that flank the T-DNA insertion site in the corresponding mutant and allowed amplification of DNA fragments from both wild-type plants and heterozygous mutants. In the T-DNA PCR reaction, a T-DNA left border (LB) primer and a gene-specific RP were used. This second PCR reaction selectively amplified the T-DNA–genomic DNA junction sequence and allowed amplification of DNA fragments from heterozygous and homozygous mutants. PCRs were performed using REDTaq ReadyMix PCR Reaction Mix (Sigma-Aldrich, Gillingham, UK) following the manufacturer’s instruction. A 1 μl aliquot of gDNA was used in a 25 μl PCR reaction, with an annealing temperature of 60°C. Primers were added at a final concentration of 0.5 μM.

### Identification of wheat *Min7* genes

Domain analysis of predicted ARF-GEF proteins in the hexaploid wheat (*T. aestivum*) was carried out using the BioMart tool in Ensembl. Initially searches were carried out for proteins that contained the Sec7_N domain (guanine nucleotide exchange factor in Golgi transport N-terminal domain; PF12783). The wheat genome assembly IWGSC RefSeq 1.0 (International Wheat Genome Sequencing Consortium, 2018) was used in this analysis. Coding sequences of eight previously identified genes comprising a small family of ARF-GEF encoding genes in Arabidopsis (Vernoud *et al.*, 2003) were also extracted using the BioMart tool in Ensembl. Multiple protein sequence alignment was carried out using ClustalW tool in Geneious v.10. For phylogenetic reconstruction, the TVM+I+G nucleotide substitution model was selected by Akaike information criterion in jModeltest v.2.1.10 (Posada, 2008; Darriba *et al.*, 2012). The maximum likelihood phylogeny was reconstructed using PhyML (Guindon and Gascuel, 2003), with the substitution model selected in jModeltest; starting tree with optimized topology, length, and rate parameters; topology searching by the best of nearest neighbor interchange and subtree pruning and regrafting; and 500 bootstraps.

### Vector construction for virus-induced gene silencing

Total RNA extracted from healthy wheat cv. Bobwhite leaf tissue was used as a template for an RT-PCR amplification of a 209-bp *TaMin7* gene fragment using primers TaMin7-2A-seg1-R (5′-AACCACCACCACCGTAAAAGGGTCGCCTCGTCAAT-3′) and TaMin7-2A-seg1-F (5′-AAGGAAGTTTAATGTTGCAAGCAAAGGCCATC-3′). This fragment was cloned in an antisense orientation into the *Barley stripe mosaic virus* (BSMV) vector pCa-γbLIC using ligation-independent cloning (Yuan *et al.*, 2011). The VIGS vector for silencing the control *TaChlH* gene was kindly provided by Prof. Dawei Li (China Agricultural University, Beijing, China). BSMV::*mcs4D*, containing a 275-nt non-coding DNA sequence), was used as a negative control (Saintenac *et al.*, 2018). To prepare the virus inoculum for wheat inoculation, the BSMVα, BSMVβ, and recombinant BSMVγ derivatives containing *mcs4D* or fragments of the *TaMin7* or *TaChlH* genes were transformed into the *Agrobacterium tumefaciens* strain GV3101 (pMP90). Agroinfiltration of *Nicotiana benthamiana* leaves was carried out as previously described (Lee *et al.*, 2015). The agroinfiltrated *N. benthamiana* leaves were harvested 5–7 d post-agroinfiltration, homogenized in 10 mM Na-phosphate buffer (pH 6.8) containing 1% Celite 545 AW (Sigma-Aldrich), and the sap was mechanically inoculated onto wheat leaves just prior to appearance of a flag leaf. Fungal inoculation of wheat spikes at anthesis was then carried out as described above.

### Quantification of gene expression and fungal biomass by quantitative real time PCR

For quantification of gene expression, total RNA was isolated from either Arabidopsis leaves or spike tissue of wheat plants using the TRIzol reagent (Thermo Fisher Scientific, Waltham, MA, USA) following the manufacturer’s instructions. To remove any traces of gDNA contamination, RNA samples were treated with TURBO DNaseI (Thermo Fisher Scientific) using methods as described by the manufacturer. The first strand cDNA was synthesized from 1 µg of total RNA in a total volume of 20 µl using the SuperScript IV Reverse Transcriptase (Thermo Fisher Scientific) and oligo (dT)_18_ primers according to the manufacturer’s instructions.

Gene-specific primers were used for RT-quantitative real time PCR (qPCR) analysis of transcript levels (Supplementary Table S1). Relative transcript levels were calculated by comparative threshold cycle (∆∆*C*_t_) and normalized to the Arabidopsis *ACTIN2* gene or the wheat *CDC48* gene (Lee *et al.*, 2014; Masachis *et al.*, 2016).

A no template control was included in each of the qPCR experiments. For quantification of fungal biomass, total genomic DNA was extracted from infected leaves at 6 d post-inoculation using DNAeasy Plant Mini Kit (Qiagen, Manchester, UK) and subjected to qPCR using the primers specific for the *F. graminearum ACTIN* gene (Supplementary Table S1). Relative amounts of fungal DNA were calculated by comparative ∆∆*C*_t_ and normalized to the Arabidopsis *ACTIN2* gene (Masachis *et al.*, 2016).

### Protein extraction and western blots

Total leaf protein preparations were made as previously described (Sainsbury *et al.*, 2009). Samples were resolved on 8% SDS-PAGE gels (Mini-PROTEAN, Bio-Rad) and transferred on to a nitrocellulose membrane (Hybond ECL, GE Healthcare). Immunoblots were performed by standard procedures using the Arabidopsis MIN7 specific antibodies at a dilution of 1:3000 (Nomura *et al.*, 2006). The blots were developed using ECL Plus Western Blotting Detection Kit and images were acquired using Odyssey Imaging System (LI-COR Biosciences Ltd, Cambridge, UK).

### Experimental design and statistical analysis

For the following experiments, GenStat for Windows (19th Edition, 2017; VSN International) was used. For Arabidopsis leaf inoculation assays, disease was quantified by expressing the diseased leaf area relative to the total leaf area. The statistical design for Arabidopsis leaf inoculation assays consisted of randomized blocks. Twelve leaves, one from each genotype, were placed onto each of eight plates. Seventy-two plants were used in total for each experiment (six plants per genotype). Six plates were used for fungal inoculation, and two plates were inoculated with water supplemented with DON to be used as a control. Three independent experiments were performed. Disease was quantified by expressing the diseased leaf area relative to the total leaf area. Mean disease levels for each genotype were compared using a multi-stratum ANOVA. Independent mutant genotypes were compared with the wild-type plants using a Dunnett’s test at the 5% (*P* < 0.05) level of significance using wild-type Arabidopsis Col-0 as the control test. GenStat (release 20.1, 2019; VSN International) was used for the statistical analyses.

*AtMin7* expression and fungal biomass determined by qPCR were compared from three biological replicates using ANOVA. Significance of differences between calculated means was determined using least significant difference (LSD) at the 5% level of significance.

For the statistical analysis of *F. graminearum* disease data, ANOVA was performed on the mean of the infected spikelets below the inoculation point out of the total spikelets calculated from the control (no virus and BSMV::*mcs4D* treated) and *TaMIN7* silenced plants of three independent experiments, and linear models were fitted using GenStat. Graphical representations were made using the ggplot2 (Wickham, 2016) package in R.

## Results

### Assembling a collection of Arabidopsis mutants with defects in membrane trafficking

To investigate whether vesicular trafficking plays a role in a compatible interaction (i.e. disease formation) between the ascomycete fungus *F. graminearum* and its laboratory host Arabidopsis, we assembled a collection of 11 mutants containing T-DNA insertions in nine immunity-associated genes regulating different vesicular trafficking pathways (Fig. 1A; Table 1). Homozygous mutants were obtained from the Nottingham Arabidopsis Stock Centre (NASC, UK), and each mutant was verified by PCR amplification using gene-specific and T-DNA-specific primers (Supplementary Table S1). Smaller rosettes were observed for three mutants (*vamp721*, *atmin7-1*, and *atmin7-2*) when compared with the corresponding wild-type Arabidopsis ecotype Col-0 plants grown under standard controlled-environment conditions. For the remaining mutants, no obvious developmental or growth defects were observed. Representative images of each mutant compared with Col-0 at two different growth stages are given in Supplementary Fig. S1.

### MIN7, an ARF-GEF protein, is required to minimize *F. graminearum* infections in Arabidopsis

To gain insight into whether mutations in any of the selected vesicular trafficking genes increase or decrease susceptibility to the virulent *F. graminearum* strain PH-1, young 6-week-old Arabidopsis plants were point inoculated with *F. graminearum* conidial suspension supplemented with the mycotoxin DON in a detached leaf bioassay (Chen *et al.*, 2006). At 6 d post-inoculation (dpi), the inoculated leaves were photographed, and disease levels were quantified by measuring the proportion of lesioned/necrotic area compared with the total leaf area by analysing the images using the LemnaGrid software module (LemnaTec GmbH, Aachen, Germany).

**Fig. 2.**
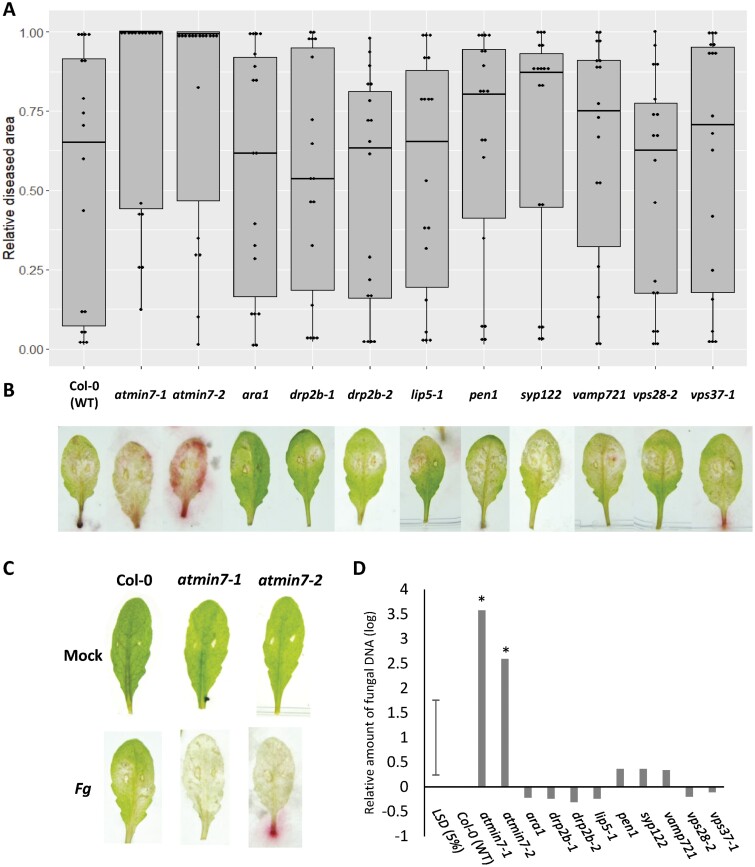
*Fusarium graminearum* infection of different Arabidopsis mutants. (A) Box-plot and dot-plot of Arabidopsis detached leaves infected with *F. graminearum*. Disease was measured as the proportion of infected leaf area compared with the total leaf area. Symbols indicate treatments statistically different from Col-0 (WT) control according to Dunnett’s test (*P* < 0.05). Data represent results from three different experiments with at least 72 leaves from 12 different plants from each mutant for each experiment. (B) The appearance of representative detached leaves 6 d after spore droplet inoculation. (C) Representative photos of leaves mock and *F. graminearum* inoculated. Mock treatments were with water supplemented with 20 µM DON. (D) Fungal biomass determined by qPCR. The relative amount of fungal DNA was calculated using the threshold cycle (ΔΔ*C*_t_) method, normalized to the Arabidopsis *ACTIN2* gene (*P < 0.05; mutant versus wild-type according to the least significant difference, LSD). *n* = 3 biological replicates.

*Fusarium graminearum*-inoculated leaves of the two independent T-DNA insertion mutants in the *AtMin7* gene (*At3g43300*) developed extensive necrotic lesions covering up to 100% of the total leaf area and showed almost complete loss of green photosynthetic tissue, while most of the remaining mutants displayed much milder disease symptoms with smaller lesions (Fig. 2A, B). Quantification of *F. graminearum* biomass by qPCR showed a markedly higher fungal burden in *atmin7-1* and *atmin7-2* mutants compared with wild-type Col-0 or any of the other analysed mutants at 6 dpi (Fig. 2C). These results strongly suggest that plants with knock-out mutations in the *AtMin7* gene are significantly more susceptible to *F. graminearum.*

*AtMin7* is a large gene of 5857 bp containing 33 exons, and is located on chromosome 3. The two loss of function mutants studied here, *atmin7-1* and *atmin7-2*, carry T-DNA insertions in exon 1 and exon 18, respectively (Nomura *et al.*, 2006). *AtMin7* is a member of a small family comprising eight genes encoding ARF-GEF proteins (Supplementary Fig. S2), which play important roles in the budding of transport vesicles from the membranes (Fig. 1A) (Steinmann *et al.*, 1999; Mossessova *et al.*, 2003). This vesicular trafficking component has been shown to contribute to resistance to the bacterial pathogen *P. syringae* pv. *tomato*, possibly through regulating the trafficking of immunity-associated cargo molecules (Nomura *et al.*, 2006, 2011).

### Expression analysis of *AtMin7* in Arabidopsis leaves infected with *F. graminearum*

**Fig. 3.**
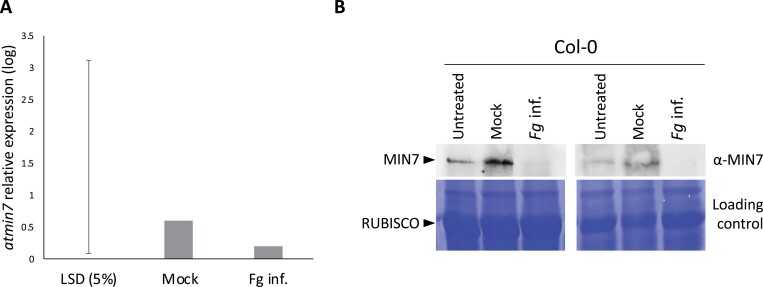
*AtMin7* expression in Arabidopsis leaves. (A) Expression of *AtMin7* in Arabidopsis leaves mock and *F. graminearum* inoculated was quantified by RT-qPCR. Mock treated leaves were inoculated with water supplemented with 20 µM DON. *AtMin7* transcript levels were not reduced in leaves infected with *F. graminearum* (*P* < 0.05; mutants versus wild-type according to the least significant difference, LSD). *n* = 3 biological replicates. (B) Western blot analyses of MIN7 in Arabidopsis leaves untreated, mock inoculated (water + 20 µM DON) and infected with *F. graminearum* (spores + 20 µM DON). Leaves were collected at 5 dpi. The endogenous MIN7 protein, detected by using the rabbit polyclonal MIN7 antibody in mock treated leaves, was absent in leaves infected with *F. graminearum*. The Coomassie stained SDS-PAGE image shown below demonstrates equal protein loading.

To understand further the mechanisms by which disruption of *AtMin7* led to Arabidopsis susceptibility to *F. graminearum*, expression of *AtMin7* was measured in wild-type Col-0 leaves inoculated with fungal spores. Transcripts levels were compared with mock-inoculated leaves (water and DON). Although considerable variation in *AtMin7* expression was noted from the fungal inoculated leaves between replicates, no significant differences were observed between the mock and *F. graminearum* treatments (Fig. 3A). Therefore, *F. graminearum* infection does not seem to suppress expression of *AtMin7* gene and/or transcript abundance during the infection cycle on Arabidopsis leaves.

We next asked if MIN7 protein levels would also remain unchanged in *F. graminearum*-inoculated leaves. To test this, MIN7-specific antibodies were used to detect the protein in Arabidopsis leaves inoculated with *F. graminearum*, mock inoculated or left untreated (Fig. 3). MIN7 was detected in untreated and mock-inoculated leaves, whereas this protein was practically undetectable in the *F. graminearum*-inoculated leaves (Fig. 3B). These results suggest that MIN7 is destabilized and/or degraded during fungal infection.

### Identification and expression analysis of candidate wheat *TaMin7* genes

**Fig. 4.**
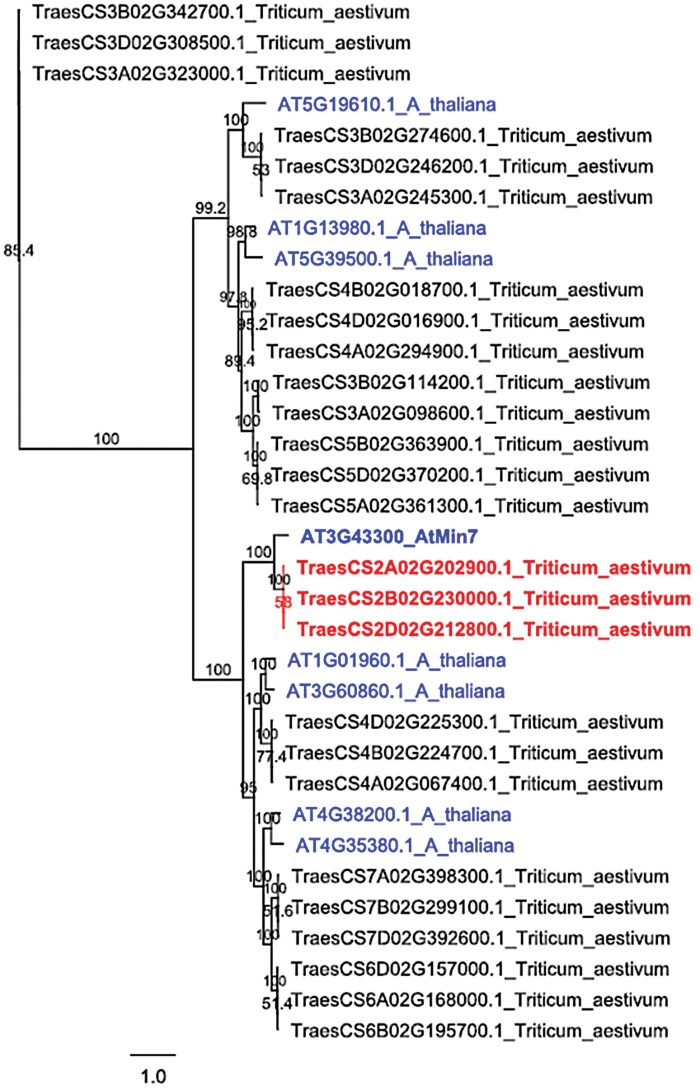
Maximum likelihood phylogenetic tree displaying representative wheat sequence orthologs of Arabidopsis ARF-GEF proteins. Node labels indicate percentage bootstrap support (500 replicates). The Ensembl Plants codes of the Arabidopsis ARF-GEF encoding genes are shown in blue regular font, with *AtMin7* accentuated in bold. The Ensembl Plants codes of wheat genes most likely coding for MIN7 are shown in red bold font, while names of all other candidate ARF-GEF encoding genes are shown in regular black font.

To identify homologs of *AtMin7* gene in wheat, a natural crop host of *F. graminearum*, we used the BioMart data mining tool available through Ensembl Plants (Smedley *et al.*, 2015). A total of 26 gene sequences were identified in the reference hexaploid wheat (*Triticum aestivum*) genome (2*n*=6*x*=42, AABBDD) by searching for genes encoding proteins containing the catalytic SEC7 domain (PF12783) characteristic of ARF-GEF proteins. We then aligned the proteins encoded by the identified wheat genes with all eight members of the Arabidopsis ARF-GEF family proteins and used the resulting multiple alignment for phylogenetic analysis. The constructed maximum likelihood phylogenetic tree revealed that the three closely sequence-related wheat proteins formed a distinct clade with Arabidopsis MIN7, suggesting these proteins represent the wheat A-, B- and D-genome-encoded orthologs of MIN7 (Fig. 4).

*TaMin7* transcript levels were also measured in wheat spikes both inoculated and non-inoculated with *F. graminearum*. Wheat spikelets were point inoculated with either water or fungal spores and tissues were collected at 5 dpi for RNA extraction and gene expression analysis using qPCR. Similar to Arabidopsis *AtMin7*, no significant differences were observed in the expression of *TaMin7* genes between mock and fungal inoculated wheat plants (Supplementary Fig. S3). Unfortunately, the lack of antibody specificity for wheat MIN7 prevented protein quantification assessments in wheat.

### Silencing the candidate *TaMin7* genes in wheat spikes enhances susceptibility to *F. graminearum*

To explore the potential function for MIN7 in the *F. graminearum*–wheat interaction, we tested the effect of silencing the three homoeologous *TaMin7* genes (*TraesCS2A02G202900*, *TraesCS2B02G230000*, and *TraesCS2D02G212800*) using BSMV*-*mediated VIGS on FHB disease development (Lee *et al.*, 2012). A 209 bp region highly conserved between coding sequences of the three *TaMin7* homoeologs was selected as a target for VIGS using the si-Fi21 software (Lück *et al.*, 2019). This target region was predicted to generate a high number of silencing-effective siRNAs (*n* = 91), and a very low likelihood of off-target silencing.

**Fig. 5.**
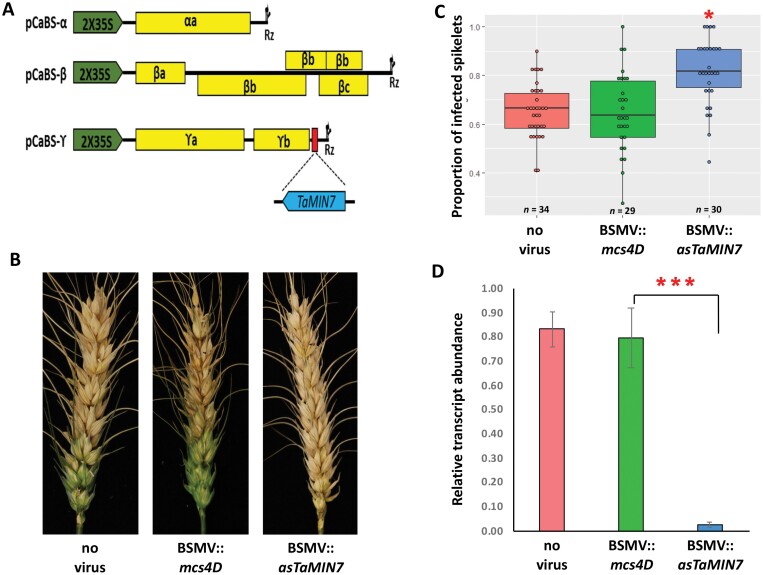
Effect of silencing *TaMin7* genes on the *Fusarium graminearum* infection in wheat spikes. (A) Schematic representation of the *Barley stripe mosaic virus* (BSMV)-derived vectors for silencing *TaMin7* (based on Lee *et al.*, 2012). cDNAs of the three BSMV genomic RNAs (α, β, and γ), each of which is required for full infection, were cloned into a binary vector, pCaBS, under the control of a double CaMV 35S promoter (2×35S) and flanked by a ribozyme sequence originating from *Tobacco ringspot virus* satellite RNA (Rz), which allows *cis-*cleavage of transcribed RNA at the 3′-end of viral genomic RNA. The RNAγ genome had been modified to include a small fragment of *TaMin7* protein-coding sequence in the antisense orientation immediately downstream of the BSMV *γb* cistron. Viral cistrons are shown as yellow rectangles. (B) Images of representative wheat spikes at 15 dpi with *F. graminearum* from virus-untreated (no virus) plants and those treated with the control (BSMV::*mcs4D*) and the VIGS construct for silencing *TaMin7* (BSMV::*asTaMin7*). (C) *TaMin7* silenced wheat plants show increased susceptibility to *F. graminearum* infection. The proportion of infected spikelets is shown as the number of visibly diseased spikelets divided by the total number of spikelets present per wheat spike below the inoculation point. For each treatment a minimum of 12 spikes were inoculated and three independent experiments were performed. *P < 0.05; *n* is number of plants. (D) RT-qPCR assessment of *TaMin7* transcript abundance in wheat plants. RNA extracted prior to *F. graminearum* inoculation and the relative abundance of *TaMin7* transcripts was calculated using the threshold cycle (ΔΔ*C*_t_) method, normalized to the wheat *CDC48* (*Cell division control 48*) gene. Error bars represent mean ±standard error of the means of three biological replicates. Significance of difference between mean transcript levels was determined using least significant differences at the 1% level of significance; ***P < 0.001.

An important factor for successful application of VIGS is the ability of the virus to infect and spread without having any deleterious effect in the host plant. Therefore, the feasibility of using the BSMV-mediated VIGS approach to induce systemic silencing in the spike tissue of wheat cv. Bobwhite susceptible to *F. graminearum* PH-1 (Brown *et al.*, 2011) was first tested by visualizing the phenotype induced by silencing the *Magnesium-chelatase subunit H* (*TaChlH*; *TraesCS7A02G480700*) gene. *TaChlH* is involved in chlorophyll biosynthesis and is often used as a visible marker in VIGS (Yuan *et al.*, 2011). The recombinant BSMV carrying a 250 bp fragment of *TaChlH* gene in an antisense orientation and BSMV::*mcs4D* harboring a non-coding DNA sequence of 275 bp were inoculated onto the flag leaves of wheat plants at the early boot stage. At 13 dpi the plants infected with BSMV::*asTaChlH* developed yellow-orange coloration of the lemma and palea of spikes indicative of the loss of chlorophyll and successful silencing of the *TaChlH* gene, whereas the spikes of plants infected with the control construct BSMV::*mcs4D* showed typical mosaic symptoms (Supplementary Fig. S4). No visible developmental abnormalities were observed in the wheat plants challenged with either of the two VIGS constructs. Moreover, similar levels of FHB disease were observed on wheat plants pre-infected with BSMV and on virus-free plants challenged with *F. graminearum*, indicating that the susceptibility to the fungus was not compromised in the virus-infected wheat (Fig. 5). These results suggest that BSMV-mediated VIGS can be used to silence genes in the reproductive tissue of wheat cv. Bobwhite and the approach appeared suitable for assessing the role of *TaMin7* during *F. graminearum* infection in wheat.

The ability of BSMV::*asTaMin7* to induce silencing of the corresponding endogenous genes was confirmed using RT-qPCR. The *TaMin7* transcripts were decreased in abundance by 77% in the spikes of the VIGS-treated plants compared with those inoculated with the negative control BSMV::*mcs4D*, and no significant difference in *TaMin7* expression levels was observed in the mock- versus BSMV::*mcs4D*-inoculated plants. To analyse the effect of *TaMin7* silencing on *F. graminearum* infection, spikes of VIGS-treated plants showing typical virus-induced symptoms were point inoculated with the fungal conidia suspension and observed for disease symptoms for 15 d after fungal inoculation. Reduction of *TaMin7* mRNA expression levels in spikes of the BSMV::*asTaMin7-*treated plants was associated with significantly enhanced susceptibility to *F. graminearum* (Fig. 5). In contrast, no effect on FHB disease development was found in mock-inoculated and BSMV::*mcs4D*-inoculated control plants (Fig. 5).

## Discussion

In this study, we have compared disease symptom formation caused by the fungus *F. graminearum* on the leaves of 11 different Arabidopsis vesicle trafficking mutant lines available in the ecotype Col-0 background. In total, eight different components of the membrane trafficking system, previously demonstrated to play important role(s) in plant defense during plant–pathogen interactions, have been evaluated (Table 1) (Gu *et al.*, 2017; Yun and Kwon, 2017; Ekanayake *et al.*, 2019). Strikingly, the two independent *atmin7* mutants were shown to be highly susceptible to *F. graminearum* infection. Similarly, when expression of the three orthologs of *AtMin7* were knocked-down in hexaploid wheat through VIGS, these *TaMin7* silenced plants displayed significantly more FHB disease formation. Quantification of *AtMin7* transcript levels in Arabidopsis and *TaMin7* in wheat revealed no evidence that *F. graminearum* infections caused a reduction in transcript abundance. Instead, immunological evidence in Arabidopsis suggested that *F. graminearum* infections cause a reduction in MIN7 protein levels.

Some bacterial and oomycete pathogens are known to have evolved effector proteins that are translocated into plant cells to promote disease formation by interfering with plant membrane trafficking pathways (Ben Khaled *et al.*, 2015). One of the vesicular trafficking genes whose role during plant disease has been previously well explored in the interactions between Arabidopsis and the bacterial pathogen *P. syringae* pv. *tomato* is *AtMin7* (Nomura *et al.*, 2006, 2011). The MIN7 protein localizes to the TGN/early endosome and is involved in endocytic recycling of plasma membrane resident proteins but it has also been hypothesized to regulate secretion (Tanaka *et al.*, 2009; LaMontagne and Heese, 2017) (Fig. 1). Mutants that lack this protein allow increased bacterial multiplication, possibly due to misregulation of membrane trafficking of the plant defense-related cargo (Nomura *et al.*, 2006, 2011). MIN7 has also been shown to contribute to the cytosol-initiated immune responses triggered by the *P. syringae* pv. *tomato* effectors such as AvrRpt2 and AvrPphB (Nomura *et al.*, 2011) and to the apoplast-initiated immune responses through an unknown mechanism by preventing apoplast water soaking and therefore presumably restricting the flow of nutrients to the bacteria (Xin *et al.*, 2016). To achieve successful disease lesion formation, *P. syringae* pv. *tomato* secretes a conserved effector protein, HopM1, which is translocated to the TGN/early endosome of its host during infection where it mediates destabilization of MIN7 followed by its degradation via the 26S proteasome (Nomura *et al.*, 2011). Here we clearly demonstrated that MIN7 also contributes to defense against the fungal pathogen *F. graminearum* because the absence of this protein in Arabidopsis resulted in a markedly enhanced disease (Fig. 2). However, whereas *atmin7* mutants displayed increased susceptibility to the *P. syringae* pv. *tomato Δcel* mutant that lacks HopM1 along with several other conserved effectors including AvrE (Nomura *et al.*, 2011), we show that these same Arabidopsis mutants are clearly and unmistakably hyper-susceptible to the wild-type strain of *F. graminearum* (Fig. 2).

Disruption of *AtMin7* may compromise trafficking of specific molecules and cargo protein. These molecules could include plant defense-related proteins and/or secondary metabolites that help minimize *F. graminearum* infection. For example, callose, a (1,3)-β-glucan polymer, can act as a physical barrier reducing fungal penetration. Both increased callose deposition and *F. graminearum*-induced callose synthase activity in the infected spikelets and rachis nodes were found to be correlated with increased disease resistance (Ribichich *et al.*, 2000; Blümke *et al*., 2014). Identification of proteins and/or other molecules transported specifically by membrane vesicles regulated by MIN7 is challenging, but comparison of vesicles cargo between wild-type and *atmin7* Arabidopsis mutants during *F. graminearum* infection could provide valuable clues to the role of MIN7 protein during fungal disease establishment.

Surprisingly, none of the other tested trafficking Arabidopsis mutants gave a distinct phenotype when detached leaves were challenged with *F. graminearum* spores (Fig. 2A). Most of the tested mutants, *drp2b-1*, *drp2b-2*, *vps37-1*, *vps28-2*, *lip5-1*, and *AtMin7*, were previously reported to be involved in immune responses to bacterial infection or bacterial pathogen-associated molecular patterns. DRP2B, VPS37, and VPS28 appear to be required for the regulation of the cell surface immune receptor FLAGELLIN SENSING 2 (Spallek *et al.*, 2013; Smith *et al.*, 2014). Disruption of LIP5 compromises basal resistance to *P. syringae* (Wang *et al.*, 2014). Therefore, it is possible that this sub-set of trafficking proteins in Arabidopsis may have specialized function(s) in response to bacterial infection, but not fungal invasion. An unexpected result was obtained with the *pen1* mutant (Fig. 2A), which encodes the first SNARE to be identified with an immune function. PENETRATION-1 (PEN1), also known as Qa-SNARE SYNTAXIN OF PLANTS 121 (SYP121), is a component of a complex of SNARE proteins that plays a role in ‘point defense’ against fungal invaders, and loss of PEN1 function in Arabidopsis leads to almost 90% penetration success of the spores of the non-adapted powdery mildew fungus *Blumeria graminis* f. sp. *hordei* (Collins *et al.*, 2003). PEN1/SYP121 forms a complex with at least four other proteins, namely SYNAPTOSOMAL-ASSOCIATED PROTEIN 33 kDa (SNAP33), Qbc-SNARE, and R-SNARE proteins VESICLE-ASSOCIATED MEMBRANE PROTEIN 721 and 722 (VAMP721 and VAMP722). We also tested sensitivity of *vamp721* to *F. graminearum* in our study and observed levels of infection undistinguished from those in the wild-type Arabidopsis plants. However, VAMP721 and VAMP722 are known to be functionally redundant. The *vamp721 vamp722* double null mutant plants could not be tested in the *F. graminearum* leaf bioassay because of severe growth defects and sometimes seedling lethality (Kwon *et al.*, 2008*b*; Yun *et al*., 2013). Finally, in this study of *F. graminearum*, the level of infection of the *syp122* mutant was comparable to that in the wild-type Arabidopsis plants (Fig. 2A). SYP122 has been shown to share an overlapping function with PEN1 during certain aspects of plant development. However, when *syp122* mutant plants were tested for resistance to *B. graminis* f. sp. *hordei*, a wild-type-like resistance response was retained (Assaad *et al.*, 2004). Collectively, these results indicate that there are differences in the function of individual vesicular trafficking proteins when defending against adapted or non-adapted fungal pathogens.

Although Arabidopsis has proven to be a very useful model organism for unraveling the key mechanisms underlying interactions with pathogens including *F. graminearum* (Brewer and Hammond-Kosack, 2015), some findings cannot be translated directly to crop plants. Hence, studies involving interactions between the pathogens and their natural host plants could provide more relevant information and facilitate exploration of new strategies for disease control in crops. Here we utilized a transient gene silencing approach (VIGS) to assess the role of *AtMin7* homologs in wheat, which is one of the most important staple food crops whose production is regularly threatened by fungal diseases including FHB. The results obtained from the analysis of the wheat–*F. graminearum* floral pathosystem (Fig. 5) are consistent with those from the study of the Arabidopsis–*F. graminearum* leaf pathosystem (Fig. 2) and provide evidence that disruption of MIN7 function in both dicotyledonous and monocotyledonous hosts and in both floral and leaf tissues compromises the plant innate immunity resulting in more severe disease.

To investigate whether *F. graminearum* utilizes similar mechanisms to the bacterial pathogen *P. syringae* pv. *tomato* to suppress host immunity and promote disease formation, the abundance of the *AtMin7* transcripts and MIN7 protein was determined (Fig. 3; Supplementary Fig. S3). In the various published whole wheat spike–*F. graminearum* transcriptome analyses, the abundance of the *TaMin7* transcripts was found to be highly variable. For example, transcriptome analysis revealed less than a 1 log2-fold change of *TaMin7* in both inoculated and non-inoculated plants in fully susceptible, moderately susceptible, or resistant cultivars (Pan *et al.*, 2018). The RT-qPCR analysis done in this study confirms that *TaMin7* transcript abundance is roughly equivalent in inoculated and non-inoculated whole wheat spikes collected at 5 dpi (Supplementary Fig. S3). However, in a previously published transcriptome study that focused on dissected out different phases of *F. graminearum* infection, *TaMin7* was down-regulated in infected tissue but only during the later symptomatic phase of disease formation (Dilks *et al.*, 2019). In contrast, western blot analysis with anti-MIN7 antibodies provided clear evidence that MIN7 protein levels decrease following *F. graminearum* infection of Arabidopsis leaves (Fig. 3). Therefore, it is tempting to speculate that *F. graminearum* may contain effector(s) functionally similar to the *P. syringae* pv. *tomato* effector HopM1 that destabilizes MIN7 to suppress host immunity and hence to promote disease. *Fusarium graminearum* does not appear to possess a homolog of HopM1, and therefore further studies would be necessary to prove or disprove the above hypothesis.

Arabidopsis MIN7 has been shown to play a role in polar localization and dynamic repolarization of the PIN (PIN-formed) efflux carrier proteins enabling the directional transport of auxin in the tissues (Tanaka *et al.*, 2013). Elevated levels of reactive oxygen species induced during stress responses in Arabidopsis affect MIN7-dependent PIN endocytic recycling resulting in increased accumulation of auxin in the affected tissues (Zwiewka *et al.*, 2019). A recent study demonstrated that higher levels of auxin are accumulated during *F. graminearum* infection in a susceptible wheat cv. Roblin compared with the moderately resistant cultivars Wuhan 1 and Nyubai, indicating that auxin may promote susceptibility to this fungal pathogen (Brauer *et al.*, 2019). However, prior exogenous application of auxin increased resistance to both floral and root *Fusarium* diseases whilst cytokinin applications increased both tissues’ susceptibility (Petti *et al.*, 2012, Haidoulis and Nicholson, 2020). Collectively, the data from these previous studies together with findings from this study form the foundations of an alternative hypothesis regarding the mechanisms employed by *F. graminearum* for achieving successful infection. That is, it is conceivable that *F. graminearum* upon infection induces temporally coordinated waves of gene expression that regulate MIN7-dependent distribution and accumulation of auxin during infection, whereas the artificial elevation of auxin levels prior to infection significantly disrupts the establishment of this subtle *F. graminearum* controlled hormonal changes. Further studies are necessary to confirm or refute this hypothesis.

In this study, the initial vesicular trafficking mutant screen was done using Arabidopsis leaf infections. This approach has many advantages but also some drawbacks. For example, could some of the interaction phenotypes and mechanisms specific to *F. graminearum* infection of wheat spike tissues have been missed by the experimental approach taken? In the last three years considerable advances have been made in the resources and technologies now available to the global wheat research community. For example, fully sequenced wheat genome and transcriptomes are available for the reference wheat genotype Chinese Spring as well as multiple wheat cultivars (International Wheat Genome Sequencing Consortium, 2018; Walkowiak *et al.*, 2020). In addition, mutant targeting induced local lesions in genomes (TILLING) populations and genome editing technologies can now be used in addition to VIGS for functional analyses in both hexaploid and tetraploid wheat species (Krasileva *et al.*, 2017). In the near future, by selecting and purifying the most appropriate lines from these new resources it should be possible to re-evaluate in both tetraploid and hexaploid wheat the various role(s) of the full spectrum of vesicular trafficking mutants against a range of diverse pathogens with different *in planta* lifestyles that also infect different host niches.

Elucidating the mechanisms by which membrane trafficking regulates plant immune responses and acquiring an enhanced understanding of the membrane trafficking components and pathways manipulated by microbial pathogens to promote disease will provide fundamental new knowledge for the development of novel methods of disease intervention.

## Supplementary data

The following supplementary data are available at *JXB* online.

Fig. S1. Representative photos of Arabidopsis mutants and wild-type Col-0 used in this study.

Fig. S2. Maximum likelihood phylogenetic tree indicating the relationship among Arabidopsis ARF-GEF encoding genes (coding sequences only).

Fig. S3. Expression analysis of *TaMin7* homoeologous genes in wheat spikes mock and *F. graminearum* inoculated by RT-qPCR.

Fig. S4. Silencing of *TaChlH* (*Mg-chelatase subunit H*) gene in wheat spikes.

Table S1. Primers used in this study for genotyping the various Arabidopsis mutants and for PCR analysis.

erab170_suppl_Supplementary_Figures_S1-S4

## Data Availability

All data supporting the findings of this study are available within the paper and within its supplementary data published online. The BSMV VIGS constructs used in this study are available on reasonable request from the corresponding author.
