# Supplementary material for: The vesicular trafficking system component MIN7 is required for minimizing Fusarium graminearum infection
Source: J Exp Bot. 2021 Apr 20;72(13):5010–23. doi: 10.1093/jxb/erab170 (PMC8364293; doi:10.1093/jxb/erab170)
Supplement: erab170_suppl_Supplementary_Figures_S1-S4 [file erab170_suppl_supplementary_figures_s1-s4.pdf]

Supplementary Figure S1. Representative photos of *Arabidopsis thaliana* mutants and wild-type Col-0 used in this study.

Six-weeks old plants. Photos taken just before the leaves were harvested for the detached leaf *F. graminearum* inoculation assay.

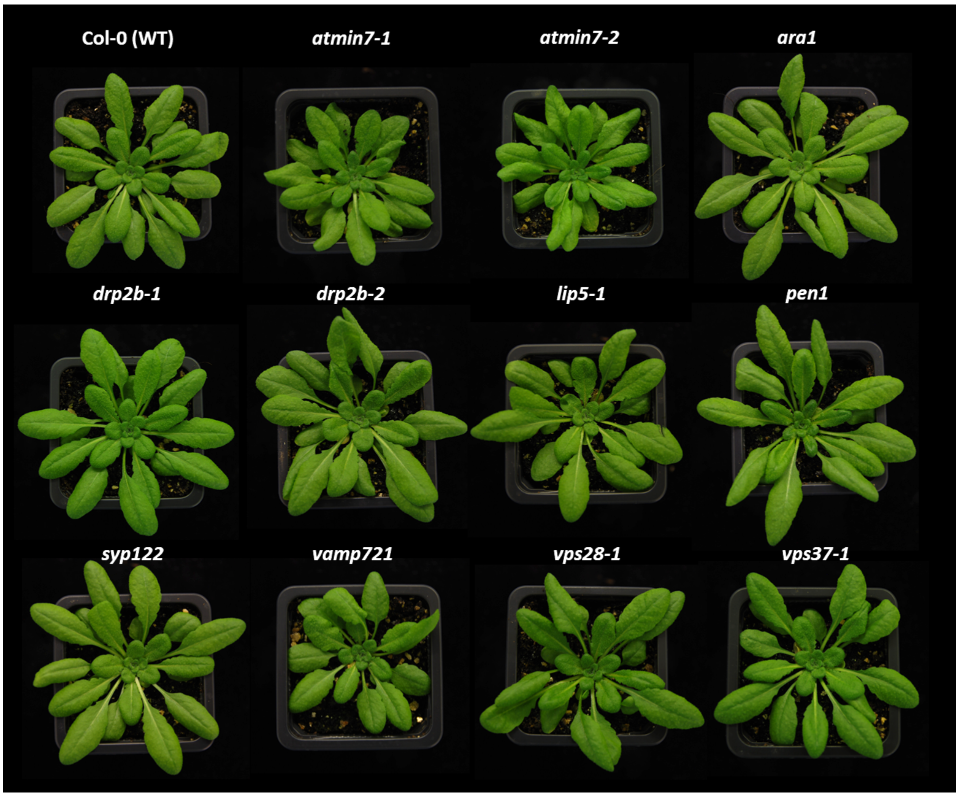

Thirteen-weeks old plants.

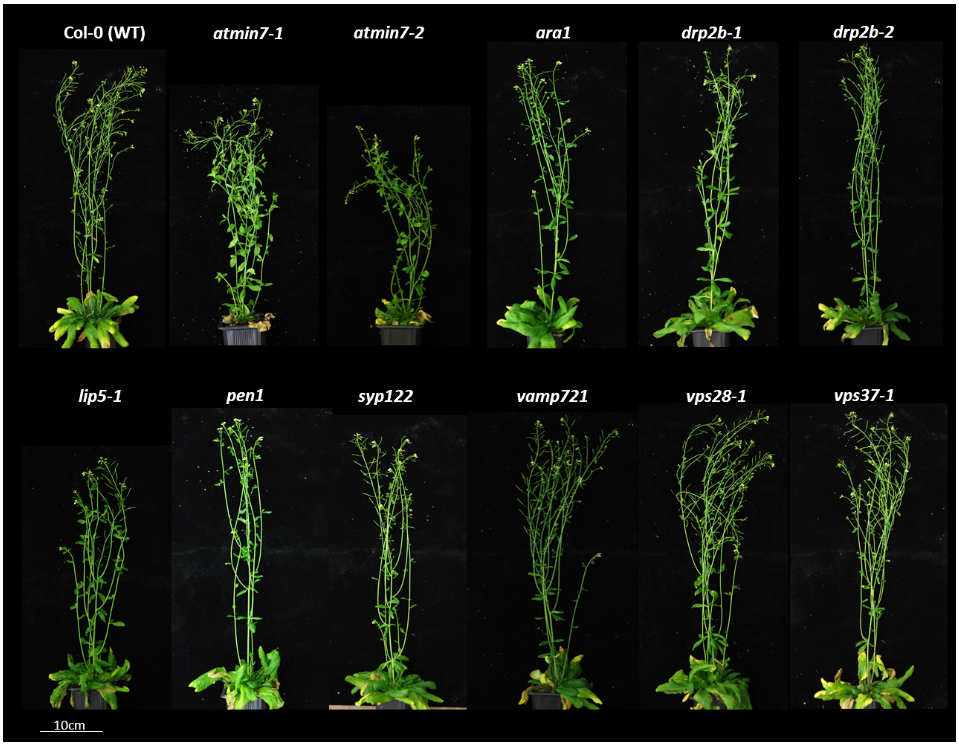

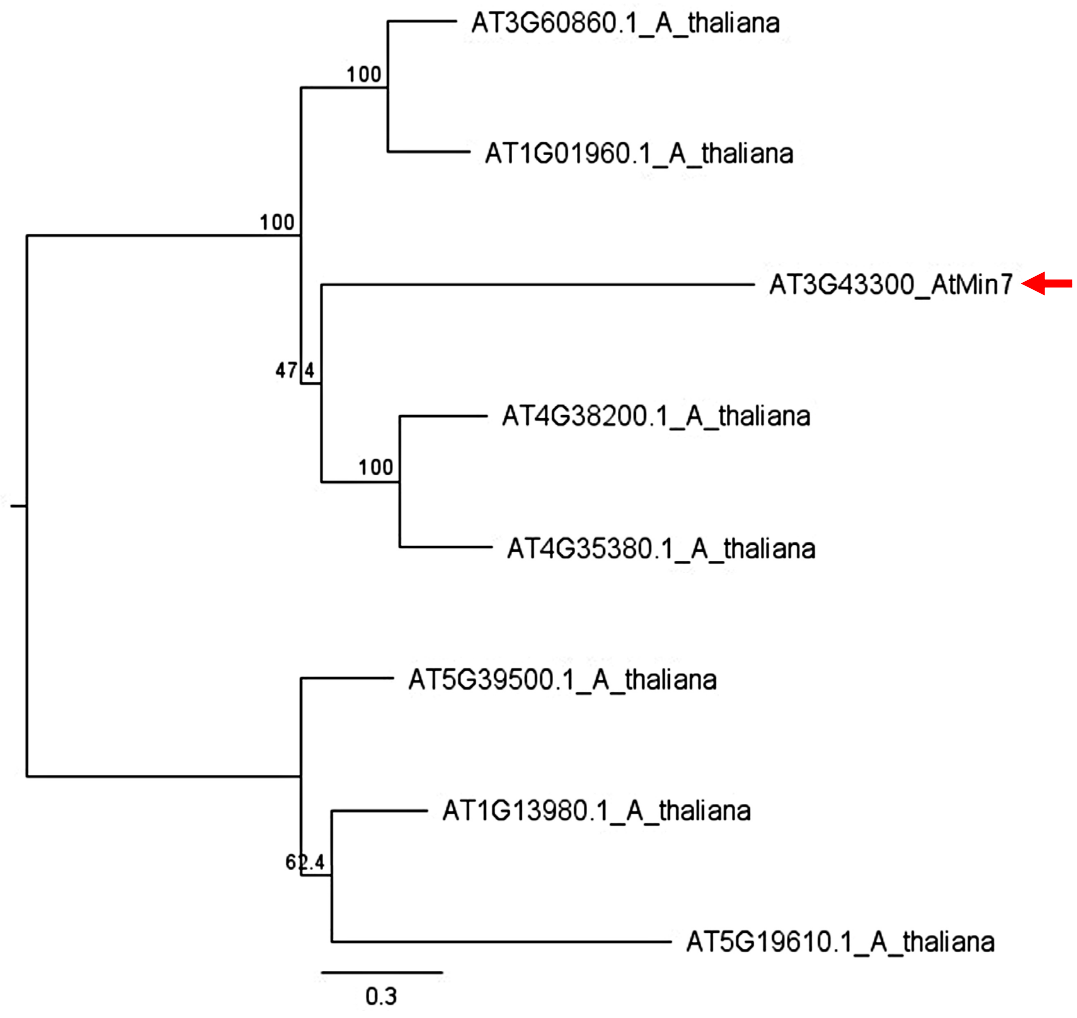

Supplementary Figure S2. Maximum Likelihood phylogenetic tree indicating the relationship among *Arabidopsis thaliana* ARF-GEF encoding genes (coding sequences only). Sequences were aligned using the ClustalW in Geneious v.10. Node labels indicate percentage bootstrap support (500 replicates). Red arrow indicates the position of *AtMin7*.

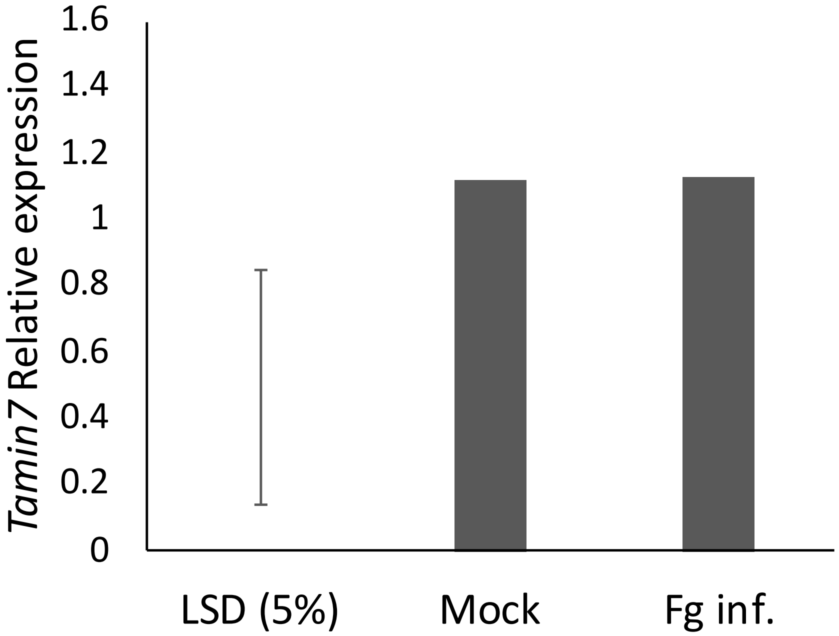

Supplementary Figure S3. Expression analysis of *TaMin7* homoeologous genes in wheat spikes mock and *F. graminearum* inoculated by RT-qPCR. Mock inoculations were carried out with water droplets. *TaMin7* transcript level was not reduced in wheat spikes collected at 5 days after *F. graminearum* inoculation ($P < 0.05$ (*); mock versus *F. graminearum* inoculated (Fg inf.) according to the least significant difference, LSD). n = three biological replicates.

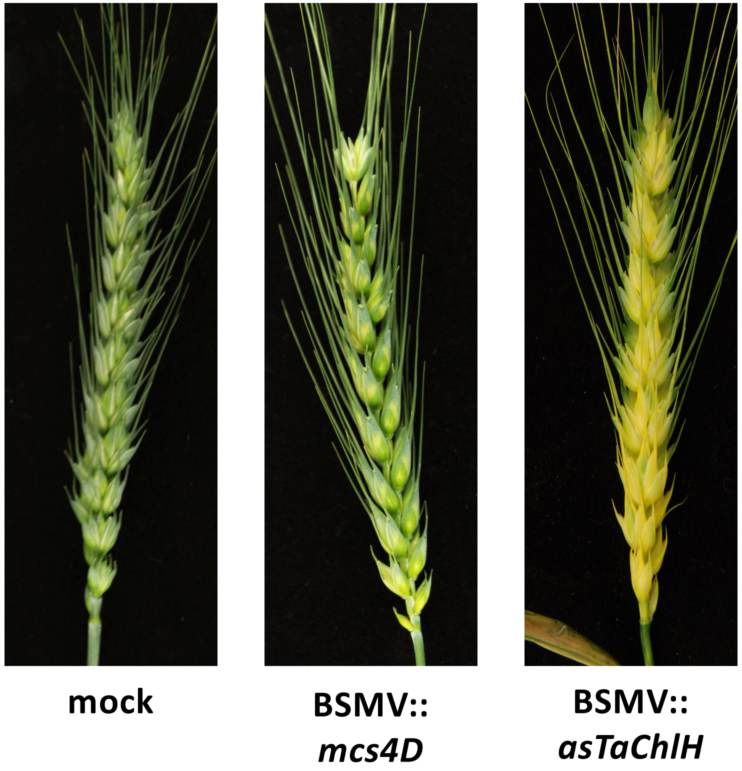

Supplementary Figure S4. Silencing of *TaChlH* (*Mg-chelatase subunit H*) gene in wheat spikes. The *TaChlH* silencing phenotype could be identified approximately two weeks after inoculation of flag leaves with BSMV::*asTaChlH* whereas those inoculated with a negative control BSMV:*mcs4D* construct and mock-inoculated plants showed no spike yellowing phenotype. This quantitative data matches the qualitative data previously reported by Lee *et al.* (2012).

Supplemental Table S1. Primers used in this study for genotyping *Arabidopsis thaliana* mutants.

Primer Name	Sequence	Application
**T-DNA_LB**	ATTTTGCCGATTTCGGAAC	Genotyping_T-DNA 'left border'
***ara1*_LP**	CCTCGCTTTTCCCAAATAATG	Genotyping_gene-specific 'left-primer'
***ara1*_RP**	TCTTCCGTCTCCTCCTCTTTC	Genotyping_gene-specific 'right-primer'
***drp2b-1*_LP**	CGAAAGGGCAGAAAAAGAAAG	Genotyping_gene-specific 'left-primer'
***drp2b-1*_RP**	ATAGCTTTGTTTGGGCATGTG	Genotyping_gene-specific 'right-primer'
***drp2b-2*_LP**	ATAGCCTAATTGGGCATCCAG	Genotyping_gene-specific 'left-primer'
***drp2b-2*_RP**	TATAGCATCGTTGTGCTGTGC	Genotyping_gene-specific 'right-primer'
***pen1/syp121*_LP**	TTGCGAGCAGCTATCTTTAGC	Genotyping_gene-specific 'left-primer'
***pen1/syp121*_RP**	GGCGGTTTTATTGAAAAGTCC	Genotyping_gene-specific 'right-primer'
***atmint7-1*_LP**	TTCTTCTCTGCTGTCAGGCTC	Genotyping_gene-specific 'left-primer'
***atmint7-1*_RP**	TTGACCAACGAATTTTTCACC	Genotyping_gene-specific 'right-primer'
***atmint7-2*_LP**	TGGAAAGTGAAATTGGTGAGC	Genotyping_gene-specific 'left-primer'
***atmint7-2*_RP**	CAAGGATTCTTCTCTGCATGG	Genotyping_gene-specific 'right-primer'
***vps37-1*_LP**	AAGAAGCTTCCTGAGGACGAG	Genotyping_gene-specific 'left-primer'
***vps37-1*_RP**	TTCGCGATTGGTATACCTGAC	Genotyping_gene-specific 'right-primer'
***vamp721*_LP**	CCCCCGTCCATTAAGAATTAAG	Genotyping_gene-specific 'left-primer'

***vamp721*_RP**	TATCAACCAAAGCTACCACGG	Genotyping_gene-specific 'right-prime]r'
***lip5-1*_LP**	ATTTATCCATCCCATCAAGCG	Genotyping_gene-specific 'left-primer'
***lip5-1*_RP**	GTTGAGAACACACACACGCAC	Genotyping_gene-specific 'right-primer'
***vps28-2*_LP**	TCAAATTAATAAAATTTCACGGTCC	Genotyping_gene-specific 'left-primer'
***vps28-2*_RP**	GACAAACGCGAAAGAGAGATG	Genotyping_gene-specific 'right-primer'
***syp122-1*_LP**	CAACTTGCGCTATTTTCTTGC	Genotyping_gene-specific 'left-primer'
***syp122-1*_RP**	TTAACTTCATCAAACCGACCG	Genotyping_gene-specific 'right-primer'
Fg_actin_F	ATGGTGTCACTCACGTTGTCC	qPCR for *F. graminearum* actin endogenous gene (forward)
Fg_actin_R	CAGTGGTGGAGAAGGTGTAACC	qPCR for *F. graminearum* actin endogenous gene (reverse)
At_actin2_F	TCCCTCAGCACATTCCAGCAGAT	qRT-PCR and qPCR for Arabidopsis actin2 endogenous gene (forward)
At_actin2_R	AACGATTCCTGGACCTGCCTCATC	qRT-PCR and qPCR for Arabidopsis actin2 endogenous gene (reverse)
AtMin7_F	CTGCATGGAGGGATTTAAAGCTGGA	qRT-PCR for Arabidopsis TaMin7 gene (forward)
AtMin7_R	TCTGAGTCACACAACCCCAGT	qRT-PCR for Arabidopsis TaMin7 gene (reverse)
TaMin7_F	ATCTTGCGGCAAAAACCAGT	qRT-PCR for wheat TaMin7 gene (forward)
TaMin7_R	ACCTGCTGAGCCACATGAAA	qRT-PCR for wheat TaMin7 gene (reverse)
TaCDC48_F	AAATACGCCATCAGGGAGAACATCGAG	qRT-PCR for wheat CDC48 endogenous gene (forward)
TaCDC48_R	CTCGCTGCCGAAACCACGAGAC	qRT-PCR for wheat CDC48 endogenous gene (reverse)
